# Factors influencing the mental health help-seeking behaviours of construction workers in Ireland: perspectives from managers

**DOI:** 10.1093/heapro/daaf210

**Published:** 2025-12-03

**Authors:** Emilie Roche, Shane O’Donnell, Noel Richardson

**Affiliations:** The National Centre for Men’s Health, Department of Health and Sports Sciences, South East Technological University, Kilkenny Road Campus, Kilkenny Road, Carlow R93 V960, Ireland; The National Centre for Men’s Health, Department of Health and Sports Sciences, South East Technological University, Kilkenny Road Campus, Kilkenny Road, Carlow R93 V960, Ireland; The National Centre for Men’s Health, Department of Health and Sports Sciences, South East Technological University, Kilkenny Road Campus, Kilkenny Road, Carlow R93 V960, Ireland

**Keywords:** suicide prevention, male-dominated industries, gender-responsive programmes, workplace health promotion, masculine norms, help-seeking attitudes

## Abstract

Men in the construction industry are at a higher risk for suicidality when compared to the general male population. While industry-specific challenges such as excessive working hours and a pressurized work environment are contributory factors, deeply embedded masculine norms within the industry can further exacerbate this risk by discouraging mental health disclosure and help-seeking. Against this backdrop, managers occupy a pivotal position to potentially transform this wider workplace culture. Their dual perspectives—both professionally as gatekeepers and personally as individuals with lived experience of key pressure points within the industry—can provide a nuanced, in-depth understanding of the sociocultural influences affecting construction workers’ help-seeking behaviours for mental health challenges. Despite this, managers’ perspectives are underexplored. Five focus groups were conducted with managers (*n* = 33) to explore their experiences of the broader cultural influences on help-seeking behaviour within the industry. Reflexive thematic analysis was used to analyse the data. Findings indicated three themes: (i) industry influences on help-seeking, (ii) navigating disclosure of mental health issues in a male-dominated industry, and (iii) negotiating support pathways. Findings highlight the interconnectedness of industry-specific and personal challenges that influence the disclosure of mental health issues and shape help-seeking behaviours among construction workers. Study findings have informed the development of a gender-responsive suicide prevention programme for the Irish construction industry.

Contribution to Health Promotion

- Highlights the interacting individual and systemic challenges that affect construction workers’ mental health help-seeking behaviour
- Explores the impact of cultural masculinities on mental health disclosure and help-seeking in male-dominated workplaces
- Demonstrates the need for gender-responsive interventions that account for masculine norms in workplace settings

Suicide rates remain consistently higher among men than among women (World Health Organization 2021), despite men having lower reported levels of suicidal ideation, suicide attempts, and diagnosed mental health conditions (Maestre-Miquel *et al*. 2021, Richardson *et al*. 2023). This ‘gender paradox’ in suicidal behaviour (Canetto and Sakinofsky 1998) has given rise to the characterization of men’s mental health as a ‘silent epidemic’ (Bilsker and White 2011) defined by a reluctance to seek help (Seidler *et al*. 2016), the underreporting of mental health conditions (Smith *et al*. 2018), and delayed mental health treatment (Smith and Hebdon 2024). These challenges are further compounded for men who adhere to more restrictive masculine norms, such as emotional suppression, stoicism, and self-reliance (Seidler *et al*. 2016). Recent research suggests that men working in male-dominated industries, typically those with over 70% male employees in the construction, mining, agriculture, information technology, transport, and utility sectors (Roche *et al*. 2016, Hulls *et al*. 2022), may be particularly at risk.

Men working in these industries are at a higher risk for depression and suicide when compared to the general male population (Roche *et al*. 2016, Tyler *et al*. 2023) and may be particularly reluctant to seek mental health support when compared to workers in other industries (Milner *et al*. 2019). While paid work can promote men’s mental health and reinforce masculine identity (Oliffe and Han 2013), harmful working conditions can undermine these benefits (World Health Organization 2022). This is evident in the construction industry, where job instability, excessive working hours, low job control, lack of social support, and a poor work–life balance are associated with poor mental health among employees (Battams *et al*. 2014, Chan *et al*. 2020, Frimpong *et al*. 2022). Moreover, the industry’s transient nature can exacerbate perceived financial insecurity among workers (Chan *et al*. 2020), a factor frequently linked with poor mental health among men (Oliffe and Han 2013). The ‘macho’ culture of the industry further undermines employee mental health. The workplace is a key setting where masculinity is performed (West and Zimmerman 1987, Connell and Messerschmidt 2005), and expectations of masculine behaviour vary across occupational groups (Reid *et al* 2018). In male-dominated industries, workers typically overemphasize traditional masculine norms such as ‘self-reliance’, which is associated with poor mental health outcomes among men (Milner *et al*. 2018). Masculinity, therefore, is a status that must be earned and constantly demonstrated and is easily lost (Vandello and Bosson 2013). As a result, workers may unintentionally uphold harmful behaviours, such as trying to ‘tough it out’ during mental health struggles, to avoid seeming ‘weak’ (Seaton *et al*. 2019). This reinforces a prevailing ‘man-up’ culture in the industry characterized by the concealment of mental health issues, reluctance to seek help, and the persistence of mental health stigma (Duckworth *et al*. 2024, Roche *et al*. 2025).

Addressing these challenges requires multilevel interventions (Roche *et al*. 2025), with managers being a key target (LaMontagne *et al*. 2014). Managers can modify employees’ exposure to harmful working conditions (St-Hilaire *et al*. 2018), influence employee attitudes towards mental health at work (Wu *et al*. 2021), and shape the wider workplace culture. This is particularly crucial in male-dominated industries, where managers can serve as role models that prioritize health and dismantle the ‘macho’ nature of industry (Roche *et al*. 2025). Additionally, they often have years of lived experience working in the industry and are attuned to the day-to-day challenges (Roche *et al*. 2025). Despite occupying such a pivotal role, managers’ perspectives remain underexplored. Existing research has documented a range of risks to workers’ mental health through systematic reviews (Chan *et al*. 2020, Frimpong *et al*. 2022), mixed-methods studies (Eyllon *et al*. 2020, Dennerlein *et al*. 2021), and prevalence studies (O’Donnell *et al*. 2024, Roy *et al*. 2024). However, there is a scarcity of research that explores the sociocultural context that shapes construction workers’ willingness to seek help for mental health challenges. While some studies have explored these challenges across different occupations within the industry (Aurelius *et al*. 2024, Hon *et al*. 2024), none have focused specifically on the perspectives of managers, despite often being the first point of contact for workers in distress (McGrath *et al*. 2023).

Managers’ dual experience supporting workers in distress and as individual’s familiar with industry pressures can potentially provide valuable insights into how workers navigate these challenges. This positions managers as key contributors to suicide prevention efforts, in line with best practice (Watling *et al*. 2022). Furthermore, discrepancies between managers’ and employee representatives’ perceptions of mental health risks in work underscores the need for exploring challenges from multiple perspectives (Houtman *et al*. 2020). The aim of this study, therefore, was to explore managers’ perspectives on the sociocultural influences on help-seeking behaviour. The study sought to address the following research question: ‘What factors influence construction workers’ intention to seek help when in distress?’. Findings will advance understanding of the contextual factors that influence mental health and help-seeking behaviours among construction workers and provide insight for designing gender-responsive interventions in the industry.

## METHODS

### Participants and procedures

This study adopted a qualitative design underpinned by a constructionist epistemology to address the principal research question. Focus groups were selected to gather data because of their utility in fostering deeper discussions that could enable a nuanced understanding of the issues affecting construction workers (Krueger and Casey 2015). Grounded in Connell’s theory of masculinities (Connell and Messerschmidt 2005), this approach offered a useful framework to explore how socially constructed masculinities may influence help-seeking behaviour in a male-dominated industry. Ethical approval was granted by the South East Technological University’s Ethics Committee (Ethical Application Number 318). The dataset analysed in this article originates from the same study reported elsewhere (Roche *et al*. 2025). While the previous publication focused on barriers and enablers of help-offering behaviour, the current article specifically investigates help-seeking behaviour, addressing a distinct research question.

Participants were eligible to take part in the study if they were over 18 years of age and employed as a manager in the construction industry. ‘Manager’ was defined as ‘persons who are in direct supervisory roles, responsible for supervision/management of one or more workers. Managers can include administrative managers or managers who are technical specialists in their field’ (World Health Organization 2022). In the current context, therefore, ‘manager’ included health and safety personnel, occupational health staff, supervisors, site foremen, mental health staff, or anyone with a responsibility for supervising workers’ health and wellbeing. Participants were recruited through leveraging existing connections with companies involved with the wider project. Participants were known to each other prior to the focus groups, and the composition of each focus group sought to include a mix of different roles within the industry. This supported varied and nuanced focus group discussions.

Key gatekeepers in each company were contacted by the lead author, the purpose of the study was explained to them, and they were asked to assist with recruitment. Study information sheets were provided to potential participants in advance. The focus groups were facilitated by the lead author who had previous experience with qualitative data collection. Prior to the focus groups, the lead author reiterated the purpose of the study, and informed consent was obtained. Each focus group lasted ∼1 h. The topic guide explored factors that influence the mental health of construction workers and the barriers to, and enablers of, help-seeking behaviour. The topic guide was developed based on previous research and in consultation with the research team (see Supplementary File S1).

### Data analysis

The focus groups were audio-recorded and transcribed verbatim. All identifying information was removed to ensure confidentiality. Reflexive thematic analysis (Braun and Clarke 2021) was used to explore patterns of meaning in the data. This analysis followed six phases, involving familiarization with the data, generating initial codes, searching for and reviewing themes, defining themes, and writing up the findings. Informal notes, field reports, and reflective journals were maintained by the lead author throughout the data collection process to support reflexivity. The lead author read and re-read the transcripts to ensure immersion with the data. Two transcripts were initially coded independently by the authors and discussed collaboratively. These discussions were not intended to achieve consensus but rather created opportunities for alternative explanations of patterns in the data. From these discussions, a set of initial codes was developed. These codes served as a starting point for deeper engagement by the lead author, who iteratively explored both semantic (surface) and latent (underlying) level meanings within the data. The analysis was not bound to a predefined coding framework but rather reflected the researchers’ active role in the interpretation of the data set. While Connell’s theory of masculinities was utilized as a lens to interpret meaning (Braun and Clarke 2024), the coding process was largely iterative, with the lead author continuously refining codes as the interpretation of data evolved. After coding all transcripts, the lead author re-read the transcripts and reviewed codes to explore further nuances and evolving interpretations. Codes were then grouped into similar patterns to generate preliminary themes. The authors engaged in regular reflective discussions regarding themes, codes, and patterns using tables in Word documents. Data collection ceased once a sufficiently rich and nuanced interpretation of the data was achieved in relation to the research question. While the study draws on the same dataset reported elsewhere (Roche *et al*. 2025), the current findings represent a distinct thematic analysis. The lead author re-engaged with the data to explore a separate research question. This involved rereading and reanalysing the transcripts with a new lens and modifying existing codes where appropriate, resulting in the development of a distinct set of themes specific to this study’s aim.

## RESULTS

Five focus groups, comprising 33 participants, were completed between January and September 2023. The sample was predominantly male (*n* = 30) and consisted of Environmental Health and Safety (EHS) staff (*n* = 19), project managers (PMs; *n* = 5), site managers (SMs; *n* = 2), site foreman (SF; *n* = 1), medic (*n* = 1), contract managers (CMs; *n* = 2), site security (SS; *n* = 1), quantity surveyor (QS; *n* = 1), and planner/scheduler (*n* = 1) all employed by five main contracting companies in Ireland. The mean age was 44.8 years (SD = 11.05), and the number of employees managed by participants ranged from 8 to 500. Some 76% of participants had undergone formal mental health training. Participants’ years of experience working in the construction industry ranged from 1 to 40 years, with many having worked in other industry roles prior to becoming managers. This allowed them to speak from personal experience and to offer personal accounts of the challenges faced by workers, alongside their contribution to broader, organizational efforts to promote help-seeking. Findings represent both managers’ personal experiences of their own past mental health challenges when working in the industry and observations on the industry-related challenges that influence help-seeking behaviours among workers more broadly. Additionally, they shed light on the complex dynamics surrounding the disclosure of mental health issues, help-seeking behaviours, and the pathways of support available to construction workers. Although one might have expected different power dynamics to emerge from the focus groups due to the variety of managerial roles, this did not materialize. Rather, participants seemed to have a shared sense of a duty of care to the workers, which took precedence over any underlying power dynamics. This was also facilitated by the time taken to create a safe and trusting setting for the discussions (Robertson *et al*. 2018).

### ‘All they seem to do is work’: industry influences on help-seeking

This theme embodies the varying industry factors that negatively impact workers’ mental health and shape patterns of help-seeking behaviours. Findings revealed that the nature of the construction industry, characterized by long commutes, long working hours, working to tight deadlines, and enduring prolonged periods away from home, posed significant challenges to workers’ mental health:

It’s a tough, tough industry, long hours, harsh working conditions and being away from home. Potentially long commutes, because you're not always going to be working on your doorstep. (FG104, EHS director, male)

This was compounded by a precarious employment system underpinned by short-term work contracts on a project-to-project basis. These working conditions contributed to a persistent sense of job insecurity and never feeling ‘settled in’ to a role. Participants highlighted a level of uncertainty and a lack of agency that came with contract work for workers. The constant threat of unemployment and loss of earnings were considered key drivers of poor mental health, as one participant reflected on their personal experiences of the issue:

Before I joined my current company, I would have bounced around with a lot of different companies doing contract work…You could be here one day and the next day you’re told to go somewhere else, it can be quite daunting. (FG405, EHS advisor, male)

Financial difficulties placed an additional toll on construction workers’ mental health. A cost of living crisis in Ireland left many workers with little choice but to work longer hours. These economic pressures, in turn, shaped workers’ willingness to seek help for mental health challenges. The volatile nature of the industry left many workers caught in a ‘trap’ of feeling the need to work excessively while often sacrificing their wellbeing in doing so:

Finance is a big thing right now with the cost of living, a lot of lads are living day to day, pay check to pay check…. (FG405, EHS advisor, male)

Notably, working excessively was actively encouraged and leveraged by the broader industry. Long working hours were seen as normalized by project developers, and workers unwilling to do so were viewed negatively. While participants acknowledged the negative impact this had on mental health, this work culture was repeatedly rationalized as simply being ‘part and parcel’ of the industry. This attitude was particularly evident among older workers, who tended to be more accepting of long and arduous working conditions. This prioritization of hard, excessive work typically aligned with more traditionally masculine beliefs, where hard physical work, worn as a ‘badge of honour’, represented an outward display of masculinity. Participants noted that efforts to maintain cultural masculine ideals on-site were key barriers to help-seeking among workers, with many opting to persevere without the need for respite:

The older people, between 40 and 55, are the people that would have traditionally worked longer hours. They tend to stay on the job longer and getting that balance [of resting] after a hard day’s work, they find it harder to do. (FG304, CM, male)

Conversely, participants highlighted that younger workers were more likely to prioritize their wellbeing. This was reflected in a reluctance to work long hours and a desire to maintain a healthier work–life balance. Despite commending young workers’ efforts to shift this culture, participants questioned whether this was an acceptable behaviour within the cut-throat practices of the industry. Thus, younger workers were continuously caught in a bind between implementing mental health promotion guidance delivered on-site and being questioned over their commitment to work by their managers:

I find that younger people don’t want to do the long hours; they want to come in at 8 a.m. and go home at 4 p.m. They probably have the right idea, but when it’s busy we’re all cursing them. (FG502, PM, female)

We’ve all said to them when they’re going, fair play to them, but they won’t last in this industry. (FG501, PM, male)

Working away from home was another key challenge, with many jobs requiring a commute and/or significant time spent away from home. With the ever-looming threat of unemployment due to short-term contracts, participants were often left in a ‘take what you can get’ mindset, which put job security ahead of their own wellbeing. One participant became visibly upset as he reflected on his own experience of being away from his family for the working week:

I’m away Monday to Friday. I hate Mondays; I don’t mind Tuesday, Wednesday, Thursday because I’m nearly home, but Monday is.. [tough]. (FG204, EHS officer, male)

Participants noted that time spent away from home was often perceived as manageable due to the strong sense of camaraderie shared among workers in the industry. This sense of solidarity was often portrayed as men working shoulder-to-shoulder and then decompressing together after a hard day’s work before returning to the ‘slog’ the next day—a seemingly positive aspect of the industry. However, on probing more deeply, it was acknowledged that, for many workers, this provided no more than temporary relief or distraction from the more challenging aspects of the industry. More typically, participants noted that the day-to-day lives of construction workers were characterized by loneliness, isolation, and boredom associated with repetitive daily routines. One participant reflected on their personal experience of these challenges:

I worked away from home for 5 years, commuting over and back Monday to Friday. And life was… it was crap, you know? I was sitting on the corner of a bed in the evening; after having the same meal you’d have every day, with my wife and two kids at home. (FG305, QS, male)

Participants described many workers being caught in a ‘catch 22’—where work pressures led to strain in other areas of their lives. While the lack of agency regarding job retention was a key challenge for those in the industry, this typically coexisted with a strong sense of masculine duty to provide for their families—despite the negative impact this had on their mental health. The demanding, unpredictable, and volatile nature of the industry had a knock-on effect on family dynamics, and participants, reflecting on their own experiences, repeatedly noted the challenges of securing employment in the construction industry that aligned with family values. Despite knowingly taking on work that came with significant personal costs, many participants rationalized their decisions, concluding that their duty of care to provide for their families outweighed any personal or emotional toll. This loss of agency, coupled with internal conflict between competing ideals of masculinity—being emotionally available partners versus embodying the stoic provider—emerged as a key driver of poor mental health and delayed help-seeking among participants:

You’re thinking about the next job. You’re hoping the next job will be closer to home, but it’s obvious that you probably won’t get that, so you take whatever is presented to you, and then the next thing, your wife is asking the question why are you going this far away? That just takes a toll on the family. (FG206, H&S officer, male)

### *‘*You give people a stick to beat you with’: navigating disclosure of mental health issues in a male-dominated industry

This theme explores the factors that influence workers’ willingness to disclose mental health issues in the construction industry. Participants reflected on the challenges of managing mental health issues in work, including navigating the impact of masculine norms and attitudes towards mental health more broadly and overcoming the constraints presented by poor mental health literacy.

There was broad consensus that men are typically less likely to discuss or seek help for mental health issues. However, this was amplified by the macho ‘man-up’ culture within the construction industry that applauded workers’ ability to endure difficult working conditions. This culture reinforced more restrictive masculine norms that, in turn, shaped negative attitudes towards help-seeking more broadly. Displays of vulnerability, in the form of expressing personal thoughts, feelings, or problems, were seen as incompatible with masculine attributes such as self-reliance, autonomy, and independence promoted by the industry. For many workers, seeking help was equated with weakness, leading to the suppression of emotions and the presentation of a brave face to the outside world. While efforts to challenge these more restrictive masculine norms on-site were ongoing, it was acknowledged that more work was needed to further address mental health stigma in the industry:

People are not inclined to show weakness. I think it’s changing but it’s still there; to show weakness in construction, you give people a stick to beat you with for want of a better word. (FG101, EHS advisor, male)

This pressure to conceal vulnerability inevitably carried over to construction workers approach to help-seeking. In instances where workers were willing to seek help from participants, this was often a secretive practice. This covert help-seeking behaviour was driven by a belief that any outward display of vulnerability posed a threat to one’s masculine status. As discussed in theme one, hard work was the embodiment of masculinity within the industry, and help-seeking did not align with cultural expectations placed on workers to be self-reliant, which served the interests and often unrealistic expectations of the industry:

There’s still the stigma that you’re weak. Even lads that I’ve helped, they don’t want anyone else to know because they see it as a weakness. (FG503, assistant PM, male)

Mental health disclosure and help-seeking behaviour remained off-limits for some, due to fears surrounding the potential negative job ramifications of each. The fickle nature of the industry meant that, for many, the benefit of seeking help did not outweigh the risk of being seen as an ‘unreliable’ worker. Against the backdrop of job and financial insecurity, construction workers were acutely aware of the need to be seen as ‘dependable’ to safeguard their future employment in the industry. This resulted in many workers opting to endure mental health challenges and conceal their emotions, rather than risk being labelled as ‘mad’:

One issue I see when people come directly to me in confidence is they’re afraid if they have a mental health issue they can’t let people know because someone is going to go back to their supervisors and they think they will lose their job because of it… Ah sure, [people think] that lad’s mad, I’m not going to bring him onto the next project. (FG203, medic, male)

Negative attitudes towards help-seeking and mental health more broadly emerged as additional barriers to disclosing mental health issues. These attitudes were often underpinned by mental health stigma in the industry. Workers’ willingness to persevere in silence through mental health distress was often validated by the surrounding culture, with the risk of word ‘getting out’ seen as a direct threat to workers’ masculine image on-site. Help-seeking patterns were often calculated to best avoid this threat, and any subsequent feelings of ‘shame’ that might be experienced through seeking support in a ‘macho’ environment where discussions around mental health were not always acceptable. Consistent with the prevailing ‘old school’ attitudes among older construction workers described in theme one, there was significant variance in willingness to discuss mental health challenges across age groups. Participants highlighted that older workers were less comfortable with discussions around mental health more broadly and were more likely to endorse traditional masculine norms that actively worked against help-seeking:

The older guys are not as susceptible to mental health issues and mental health as the younger guys, because I think certainly when I was going to school, mental health was not …. I mean, if you admitted you had mental problems 20 years ago, you were weak. (FG101, EHS advisor, male)

Poor mental health literacy was repeatedly cited as a more fundamental barrier to construction workers seeking help. There were numerous accounts of workers having limited understanding of the signs or symptoms of mental health conditions. Importantly, participants noted that workers were particularly unaware of the mind–body connection and often misinterpreted physical symptoms of stress and anxiety. Failure to recognize these symptoms often contributed to more severe distress in the longer term, further exacerbating psychological and physical symptoms. This had knock-on effects on knowing when and how to seek support:

I saw chest pain from a couple of individuals who would come in feeling really anxious, nearly at a panic attack level, and they’re not identifying that this is actually a panic attack and then they don’t know how to subsequently manage that. (FG203, on-site medic, male)

### *‘*Talking is key’: negotiating support pathways

This theme highlights how navigating informal support pathways—especially rooted in personal relationships and emotional connection—plays a vital role supporting construction workers’ mental health. It sheds light on where and to whom construction workers turn to for support and documents ongoing efforts to promote mental health in the industry more broadly.

Given the challenges to mental health disclosure and help-seeking within the industry described in theme two, participants placed strong therapeutic value on the idea of returning ‘home’. ‘Home’ represented a crucial source of emotional relief and support, a refuge to reconnect with family, partners, and children. However, participants acknowledged that it was not a fortress and therefore not immune to the intersecting and destabilizing pressures of the industry described in theme one. There was broad agreement that such challenges placed considerable strain on intimate relationships, with potentially catastrophic consequences in terms of loss of the most primary support system. One participant reflected on their own experience of these pressures:

I don’t usually break radio silence on this, but from a personal point of view, I am divorced, due to the downturn of the construction industry. I lost everything, yeah. Nearly my children as well. (FG106, SF, male)

Loss in this sense not only signified the breakdown of relationships and familial ties, but it also represented the erosion of a psychological ‘safe haven’ in which workers could seek support. The removal of these safety nets left participants at risk of loneliness and despair, no longer able to lean on the emotional support once offered by the familiar setting of ‘home’. The dismantling of this scaffolding of support was a significant impediment to workers’ capacity to deal with mental health challenges, as they faced up to the uncertainty, isolation, and the painful realization of being alone. One participant shared their own experience of navigating this loss of support:

Personally, for myself anyway, I would be an overthinker. When I separated from the kids’ mother, the hardest thing I found dealing with was coming home to an empty house. You kind of realize, I’m on my own here. (FG103, EHS officer, male)

While there was broad agreement that peer support went some way towards filling the void associated with being away from familiar supports, it was acknowledged that the type of support offered within a predominantly male ‘macho’ environment rarely extended to conversations around mental health concerns. Therefore, the absence of more intimate personal connection while working away from home, coupled with the culture of stoicism within the industry, often left workers with few ‘acceptable’ avenues to seek support, leaving many mental health issues going unchecked:

One thing I’ve noticed throughout my career is people working away from home and working away from their families; there’s no outlet. All they seem to do is work because they don’t have anyone close or personal to share any problems or issues with, so it just gets bottled up. (FG304, CM, male)

Repeated references to an alcohol culture in the construction industry were seen as a further challenge to accessing meaningful support. While it was acknowledged that this culture was shifting, participants noted that alcohol use was still considered an acceptable coping strategy, particularly during periods of boredom, isolation, and loneliness associated with being away from home. Even where a strong sense of camaraderie existed among workers, participants noted that engaging in maladaptive coping strategies was still seen as a more palatable option for managing mental health concerns than the socially ambiguous and often stigmatized practice of opening up to workmates:

Some people go to the bottle, you know, they’re not at home, they’re in their B&Bs, there’s nothing to do. I mean, it’s nice to have a drink but when you’re by yourself in a hotel room for 5 days, I don’t think that’s going to help [your mental health]. (FG203, medic, male)

Despite these barriers, participants welcomed the fact that there were ongoing efforts to promote help-seeking among construction workers across the industry. These supports included employee assistance programmes (EAPs), confidential phone services, mental health first aiders on-sites, collaborations with charitable organizations, wellness initiatives, and community events. Ongoing attempts to raise awareness of mental health included the provision of mental health supports on-sites using internal talks, workshops, and posters. There was broad agreement that the strongest enabler of seeking any kind of support was through normalizing conversations around mental health. One participant shared their own experience when accessing support, highlighting that at their core, workplace mental health initiatives should aim to facilitate discussions around mental health challenges that would, in turn, shape a broader workplace culture conducive to disclosing vulnerability.

I was in the process of trying to save my marriage, and I was in marriage counselling and, my wife at the time, just got up and left. Okay, that was that. I stayed with the chap for around 10 sessions. Talking to that chap completely put me back on track, to where I could make a plan, and where I was able to keep driving forward. What I’m trying to say is talking is key. Talking is key…. (FG106, SF, male)

Despite advancements in mental health promotion initiatives on-site, the desire to maintain a masculine bravado coupled with the need to be seen as reliable contributed to a sense of suspicion towards engaging with available company supports. There was a clear acknowledgement that to promote help-seeking among workers, more needed to be done to challenge mental health stigma and to allay fears around confidentiality at the organizational level. Participants emphasized that leadership plays a crucial role in fostering this openness. A key strategy in achieving this resided in managers’ willingness to share their own experiences of help-seeking to reassure workers that it was an acceptable practice:

We have a company employment scheme. I’ve used it before for legal reasons, and I told [employees] that I have used it in the last 3 or 4 years, and that the company doesn’t know what we’re ringing about, and you just keep reassuring [employees] and saying they are anonymous. (FG503, assistant PM, male)

This willingness to share personal experiences of poor mental health and help-seeking was seen as a key enabler for others to access supports. Real-world stories were cited as powerful tools in establishing empathy, rapport, and mutual understanding. This approach also leveraged camaraderie among workers and promoted a sense of solidarity of men working together and building a safe community to discuss mental health. This was highlighted as being fundamental to building strong support pathways for workers. Additionally, the use of role models with lived experience of poor mental health was seen as being useful both to encourage help-seeking and to challenge traditional masculine ideology by creating permission for and normalizing conversations around mental health among workers:

About 2 or 3 years ago I told my story of when I was in a bad place, and I remember at the very start I asked the question has anyone ever suffered from poor mental health? And no one put up their hands; I told my story and afterwards I asked again, and 70% of the room put up their hands. (FG403, EHS manager, male)

Although accessing more formal support was deemed to be important, findings revealed that the availability of simple, informal supports also had a critical role to play. The use of nonmedicalized language when promoting conversations around mental health was seen as key. There was agreement that much work was needed to further reduce stigma to foster long-term change:

Men in particular would feel a weakness in saying they have problems, but then they have to say “I’ve got a mental health issue”, it’s just that language, it’s not nice. (FG305, QS, male)

## DISCUSSION

Against a backdrop of high suicidality within the construction industry, the aim of this study was to explore, through the lens of construction industry managers, the factors that influence construction workers’ mental health help-seeking behaviours. Findings shed light on the intersection of industry and personal challenges that impact construction workers’ approaches to seeking help. Managers are strategically positioned to offer both personal and professional perspectives to the study’s central research question. Participants in this study held a unique vantage point, in many cases both as construction workers with lived experience of dealing with challenges to their own mental health and as key gatekeepers for workers in distress. This enabled a nuanced understanding of the broader determinants of poor mental health and help-seeking practices in the industry. Findings offer insight into the challenges faced by construction workers, related to, and contextualized through, the lived experience of industry managers. They also offer organizational-level perspectives on the barriers to and enablers of help-seeking more broadly and the challenges relating to current health promotion efforts in the industry. Findings offer a pragmatic, dual perspective that informed the development of a bespoke suicide prevention programme for managers in the industry.

Findings reveal the intricate and interconnected challenges to construction workers’ mental health and how they relate to help-seeking behaviours. Industry-related challenges such as long working hours, lengthy commutes, financial insecurity, poor work–life balance, and transient work contracts were repeatedly identified as key contributors to poor mental health, (Milner *et al*. 2018, Chan *et al*. 2020, Newaz *et al*. 2022) and significantly shaped workers’ willingness to seek help for mental health challenges. Job insecurity and the precarious employment conditions within the industry, coupled with the ‘man-up’ culture (Blake *et al*. 2023), create an environment where mental health issues are often concealed. This was typically due to fear of job loss or appearing ‘weak’ or unreliable (Stratton *et al*. 2018, Rutherford *et al*. 2024). Findings suggest that workers’ help-seeking practices are shaped by the perceived economic consequences of mental health disclosure (Rutherford *et al*. 2024). This was particularly evident in the context of the social climate in Ireland and a rapid rise in the cost of living (Collins 2023). More worryingly, findings revealed that these challenges were frequently capitalized on by the broader industry, by enabling a culture of overwork, stoicism, and self-reliance. This highlights the need to consider the broader socioeconomic context when designing health promotion approaches for specific cohorts (Kortum and Leka 2014), alongside organizational strategies to better support workers’ wellbeing, such as limiting excessive overtime (Jenkin *et al*. 2024).

Findings highlight that men are at a particular risk for poor mental health and suicidality during prolonged separation periods involving the loss of sources of emotional support (Kõlves *et al*. 2010). Participants emphasized the crucial therapeutic value of personal relationships and the mental health implications of losing familiar support systems. While male-dominated industries have the potential to provide alternative support pathways by leveraging camaraderie on-site (Hanna *et al*. 2020), this is often undermined by dominant masculine norms in the industry that discourage help-seeking. Seeking support in the industry was often viewed as a threat to maintaining a masculine image on-site, with help-seeking viewed as something shameful, covert, and secretive. A breadth of research has linked restrictive masculine norms such as emotional suppression and self-reliance with a reticence to seek help when in distress (Galdas 2015, Seidler *et al*. 2016, Gough and Novinka 2020; Hanna *et al*. 2020, Piatkowski *et al*. 2024) and complements the broader antecedents of help-seeking at work related to environmental factors identified in Chen *et al*.’s (2023) integrative framework. Findings also complement the view of ‘manhood’ as precarious, with masculine status as something that is earned and easily lost (Vandello and Bosson 2013), particularly in sectors that may inadvertently perpetuate ‘masculinity contests’ by reinforcing norms of strength, stamina, and overwork (Berdahl *et al*. 2018).

In many instances, mental health disclosure was seen as misaligned with wider cultural norms. This resulted in a preference among workers to maintain a stoic image and ‘tough it out’ during times of distress, rather than engage in the ‘socially risky’ act of seeking help (Vandello and Bosson 2013). Maladaptive coping, particularly in the form of excessive alcohol consumption, was considered a more socially acceptable alternative to formal help-seeking. These findings reflect broader cultural norms in Ireland where alcohol use is deeply tied to masculine identity (Darcy 2019). Participants describe a culture where hard, physical labour followed by a visit to ‘the pub’ to decompress is normalized (Tilki 2006). Therefore, the pub is considered a socially acceptable space for solidarity while simultaneously reinforcing maladaptive coping mechanisms. Findings further complement broader research linking adherence to traditional masculine norms, such as emotional suppression, with increased use of alcohol as a form of escapism (Blisker *et al*. 2018). This is also in keeping with Laoire’s (2005) examination of the historical and cultural roots underpinning the ‘strong, silent’ Irish male archetype and how the suppression of vulnerability is deeply ingrained in the construction of Irish masculinities. Furthermore, poor mental health literacy, discomfort around disclosing personal information, and fears around confidentiality were identified as additional barriers to seeking support. Many men were unaware of or misinterpreted the common signs of mental health disorders (Gibbons *et al*. 2015, O’Donnell *et al*. 2025). Additionally, a level of cynicism around accessing workplace support services such as the EAP and confidential phone services (Rutherford *et al*. 2024) was highlighted. EAPs have previously been criticized for not accommodating men’s specific needs (Boettcher *et al*. 2019) and may be unsuited to the specific needs of construction workers, underscoring the importance of implementing gender-responsive interventions that reflect the unique context of the construction industry (Gough and Novinka 2020).

Overall, findings align with recommendations to promote organizational help-seeking by creating a safe environment for mental health disclosure. This can be achieved through normalizing mental health conversations, acknowledging employees’ reservations around engaging with company supports, and building trust between management and employees (Rutherford *et al*. 2024). Within the male-dominated context, findings complement broader recommendations that men’s mental health initiatives ought to be grounded in existing supportive communities that provide alternative safe spaces for men to engage in discussions around mental health (Robertson *et al*. 2018). Male-dominated industries can act as a familiar and socially acceptable environment to promote ‘shoulder-to-shoulder’ interventions (Sharp *et al*. 2022), on the condition that masculine norms are acknowledged, engaged with, and reframed. Additionally, these settings can provide crucial alternative support pathways for workers—especially for those who may otherwise rely on partners as ‘health advisors’ (Oliffe *et al*. 2011). There was strong support for this type of approach among participants, with many demonstrating a willingness to challenge and rework more restrictive cultural norms in the industry.

Findings highlight that role models willing to share real-world stories of mental health challenges played a key role in facilitating these conversations to promote help-seeking and reduce stigma, consistent with a recent systematic review of interventions in male-dominated industries (Roche *et al*. 2024). Such interactions play a key role in enhancing relational (e.g. trust and accessibility) and leader-related (e.g. support and respectful engagement) factors that enhance help-seeking in work (Chen *et al*. 2023). By granting ‘permission’ to openly discuss mental health issues among workers, role models can normalize conversations around mental health (Gough and Novinka 2020) and dismantle the ‘macho’ culture by reframing help-seeking as a sign of strength (Sagar-Ouriaghli *et al*. 2019). Therefore, future psychoeducational training should include information on the signs of mental health conditions, clearly outline the limits of confidentiality, and educate workers around support services available to enhance suicide literacy and knowledge of help-seeking pathways. Participants’ personal accounts of mental health challenges highlight the importance of developing interventions that are cognisant of ‘cross-pressure’ and account for the impact of employee disclosure on managers’ own wellbeing (Martin *et al*. 2018). Therefore, future interventions should include dedicated information for managers in maintaining their own positive mental health. Such interventions must be gender responsive and designed to reflect construction workers’ unique needs and preferences to enhance acceptability. Similar training programmes have been shown to reduce stigma and increase suicide and mental health literacy in these industries (Gullestrup *et al*. 2023).

Crucially, ongoing industry efforts to promote mental health must counter the perception among workers that by engaging in such initiatives or by accessing supports, their commitment to their work was somehow in question. This highlights the urgency of embedding health promotion strategies within the wider workplace context through establishing organizational-level mental health policies and procedures to promote a psychologically safe environment for the disclosure of mental health issues (Jenkin *et al*. 2024). Health promotion initiatives must align with wider industry efforts aimed at addressing the broader structural determinants of poor mental health, such as provisions for guaranteed hours, stronger union representation, and improved communication surrounding potential job loss (Jenkin *et al*. 2024). This requires a multifaceted approach to help-seeking that extends beyond individual behaviour, encompassing industry-wide initiatives to normalize conversations about mental health, reduce stigma, and develop industry-wide standards to facilitate a wider culture change around mental health (Rutherford *et al*. 2024). It is recommended that future trainings utilize a codesign and collaborative approach with construction workers during the development of interventions to promote help-seeking (Galdas *et al*. 2023). These approaches engage men as equal partners and empower workers to shape interventions that reflect the reality of their work context. This type of approach has been recommended for male-dominated industries and has been shown to increase the acceptability of training materials (Greiner *et al*. 2022).

## LIMITATIONS

There are several limitations to consider when interpreting the findings. Study participants were managers in the construction industry. Therefore, findings are not generalizable to all construction workers. Future research may wish to explore a similar research question across a more diverse range of occupations on-site. While the current study sheds light on the challenges affecting construction workers’ mental health as it relates to help-seeking, more research is needed to explore these challenges and the intersectionality of these risks—particularly relating to help-seeking behaviours across age cohorts. This deeper understanding would support the development of tailored programmes that address the unique needs of specific cohorts within the industry.

## CONCLUSION

This study sheds light on the challenges impacting construction workers’ mental health as it relates to help-seeking and accessing supports. Efforts to promote workers’ mental health and help-seeking must be rooted in a broader, industry-wide approach. Future training for the industry would benefit from combining individual-level approaches with broader organizational efforts to address the wider determinants of poor mental health through industry-wide policies and guidelines. Drawing on Connell’s theory of masculinities (Connell and Messerschmidt 2005), the study highlights how dominant masculine norms continue to inhibit help-seeking behaviours in the industry, particularly among older workers. As such, gender-responsive interventions that align with the preferences and lived experience of construction workers are essential. The findings provide a potential road map for designing such interventions and have informed the development of a suicide prevention training programme for the construction industry in Ireland.

## Supplementary Material

daaf210_Supplementary_Data

## Data Availability

The data underlying this article cannot be shared publicly due to privacy and ethical considerations related to the sensitive nature of the study. However, the data will be shared upon reasonable request to the corresponding author.
