# Supplementary material for: Factors influencing the mental health help-seeking behaviours of construction workers in Ireland: perspectives from managers
Source: Health Promot Int. 2025 Dec 3;40(6):daaf210. doi: 10.1093/heapro/daaf210 (PMC12673263; doi:10.1093/heapro/daaf210)
Supplement: daaf210_Supplementary_Data [file daaf210_supplementary_data.docx]

| **Primary Questions** | **Follow up/Prompts** |
| --- | --- |
| 1. Could you tell me about some of the common issues that affect construction workers’ mental health? | What ways might you notice someone is in distress?  Explore industry specific factors |
| 1. Could you tell me about some of the existing supports in the construction industry that you are aware of? | - Are they useful/accessible? - What do you think of them? - Do people use them, why/why not? |
| 1. What stops construction workers from seeking help for their mental health? | Stigma, masculinity, practicalities, transient work, workload? |
| 1. Have you ever experienced an employee coming to you about their mental health, or have you ever reached out to an employee about their mental health? Could you tell me a bit about that? | - What did you do, how did you find it? - Was it easy or difficult? How did you feel? - What did you notice that made you reach out |
| 1. What are some of the challenges you face in offering help to employees around their mental health?   (Both as a manager and as a construction worker more generally?) | What is difficult? skills, confidence, motivation, macho culture, time constraints.  What might stop you intervening? concerns around intervening.. based on past experience were the concerns validated? |
| 1. Do you see it as part of your role to offer help to employees with mental health difficulties? Would you know what to do or where to go?   Competent or confident to offer help? Around what to say, where to signpost? | - Is there training for this, procedures in place to follow? - Is it seen as a priority in the industry? |
| 1. In your experience, what works well with regard to supporting employees around their mental health? | - What is the best way of communicating with employees around mental health? - What would construction workers respond best to? |
| 1. What would make it easier for you to support employees around their mental health? | What do you feel you need? What support do you need to support others?  Training/policies?  Culture of industry, outside the individual |
| 1. From your experience of dealing with this, what do construction companies need to do more of to support mental health and prevent suicide of employees   (Ways the wider workplace can support gate keepers to offer help) | - What type of supports do you feel work well? - What are the challenges for implementing these supports for the industry? - What positive aspects of your company could be used to implement supports? |
| 1. Is there anything you would like to add that I haven’t covered with regard to supporting employees around mental health or supporting employees to seek help? | - Any other suggestions for training/resources etc. |
